# Asymptomatic ratio for seasonal H1N1 influenza infection among schoolchildren in Taiwan

**DOI:** 10.1186/1471-2334-14-80

**Published:** 2014-02-12

**Authors:** Ying-Hen Hsieh, Chen-An Tsai, Chien-Yu Lin, Jin-Hua Chen, Chwan-Chuen King, Day-Yu Chao, Kuang-Fu Cheng

**Affiliations:** 1 Department of Public Health, China Medical University, Taichung 40402, Taiwan; 2 Institute of Biostatistics, China Medical University, Taichung 40402, Taiwan; 3 School of Public Health and Biostatics Center, Taipei Medical University, Taipei, Taiwan; 4 Department of Agronomy, Biostatistic Divison, National Taiwan University, Taipei, Taiwan; 5 Institute of Epidemiology and Preventive Medicine, College of Public Health, National Taiwan University, Taipei 10617, Taiwan; 6 Graduate Institute of Veterinary Public Health, National Chung Hsing University, Taichung 40227, Taiwan

**Keywords:** Seasonal influenza, H1N1, Asymptomatic ratio, Asymptomatic infection, Taiwan, Symptoms

## Abstract

**Background:**

Studies indicate that asymptomatic infections do indeed occur frequently for both seasonal and pandemic influenza, accounting for about one-third of influenza infections. Studies carried out during the 2009 pH1N1 pandemic have found significant antibody response against seasonal H1N1 and H3N2 vaccine strains in schoolchildren receiving only pandemic H1N1 monovalent vaccine, yet reported either no symptoms or only mild symptoms.

**Methods:**

Serum samples of 255 schoolchildren, who had not received vaccination and had pre-season HI Ab serotiters <40, were collected from urban, rural areas and an isolated island in Taiwan during the 2005–2006 influenza season. Their hemagglutination inhibition antibody (HI Ab) serotiters against the 2005 A/New Caledonia/20/99 (H1N1) vaccine strain at pre-season and post-season were measured to determine the symptoms with the highest correlation with infection, as defined by 4-fold rise in HI titer. We estimate the asymptomatic ratio, or the proportion of asymptomatic infections, for schoolchildren during the 2005–6 influenza season when this vaccine strain was found to be antigenically related to the circulating H1N1 strain.

**Results:**

Fever has the highest correlation with the 2005–06 seasonal influenza A(H1N1) infection, followed by headache, cough, vomiting, and sore throat. Asymptomatic ratio for the schoolchildren is found to range between 55.6% (95% CI: 44.7-66.4)-77.9% (68.8-87.0) using different sets of predictive symptoms. Moreover, the asymptomatic ratio was 66.9% (56.6-77.2) when using US-CDC criterion of fever + (cough/sore throat), and 73.0 (63.3-82.8) when under Taiwan CDC definition of Fever + (cough or sore throat or nose) + ( headache or pain or fatigue).

**Conclusions:**

Asymptomatic ratio for children is found to be substantially higher than that of the general population in literature. In providing reasonable quantification of the asymptomatic infected children spreading pathogens to others in a seasonal epidemic or a pandemic, our estimates of symptomatic ratio of infected children has important clinical and public health implications.

## Background

Influenza is one of the most common upper respiratory infectious diseases in humans, especially in children, although it is generally known that influenza accounts for only a proportion of the disease burden caused by respiratory virus, as respiratory syncytial virus (RSV) and para-influenza, account for a substantial proportion of these infections. Schoolchildren form an important community-based influenza epidemic sentinel group because influenza is a common disease among children. Studies have reported attack rates of 28-43% among school-aged children [1].

Children have also been found to shed virus earlier, for up to six days before the illness begins, and for a longer time period once they are infected [2-6]. However, among these reports, few studies used serological and community-based study design to evaluate the impact of influenza virus infection on schoolchildren. It is widely believed that asymptomatic cases and asymptomatic infections do occur regularly in both seasonal and pandemic influenza and is an important aspect of the epidemiology of influenza, including the past 2009 pandemic H1N1 influenza (pH1N1) [7]. Hence it was often modeled accordingly in many modeling studies [8-11] and shown to possibly impact the validity of the results. Moreover, the asymptomatic ratio (or frequency of asymptomatic infection) is also a critical parameter for public health purpose of interventions involving contact tracing. Epidemiologic studies also suggest that the natural history of influenza virus infection might differ for children (or for elderly), although no such data exist [6]. Moreover, some studies have shown that schoolchildren may play an important role in household transmissions (e.g., [12-14]). However, a recent study on age-specific timing of laboratory-confirmed influenza infections using laboratory-confirmed data from Canadian communities has cast doubt on the hypothesis that younger school-age children actually lead influenza epidemic waves [15].

Furthermore, a recent community-based household study [16] in central Taiwan on antibody response against seasonal H1N1 and H3N2 vaccine strains in schoolchildren receiving only pH1N1 monovalent vaccine revealed seroconversion rate of 32.8% to seasonal H3N2 vaccine strains, suggesting that the wild-type influenza virus, especially H3N2, might have co-circulated in the community, as co-circulation of the 2009 pH1N1 and seasonal strains had also been reported elsewhere [17]. More importantly, the results on the seroconversion rate of H3N2 vaccine strain observed in schoolchildren not receiving TIV and had few clinical symptoms raise the question that children might acquire asymptomatic or subclinical infection, and perhaps play a significant role as the major disseminators in the spread of seasonal influenza [18,19].

Moreover, in a related study in Taiwan [20] serological evidence indicates significant seroconversion of antibodies to the pH1N1 virus with an HI titre of 1:40 by September–October in 2009 among 306 schoolchildren tested, further highlighting the importance of children as asymptomatic transmitters of influenza in households.

The focus of this paper is to determine which symptoms are the most effective clinical predictors of influenza and the asymptomatic ratios of human influenza viruses in schoolchildren populations in Taiwan using seasonal influenza sero-epidemiologic data of schoolchildren in Taiwan of 2005–2006 winter influenza season, in order to understand asymptomatic influenza infection among schoolchildren.

Influenza infectious symptoms are usually associated with fever, headache, cough, sore throat, running nose, myalgia, malaise, and rhinitis. Infected children can also display signs of otitis media, nausea and vomiting. However, studies reporting on proportion of seroconversion cases that had symptoms vary widely in their definition of symptoms or clinical predictors (see, e.g., [6,21-24]). The US Centers for Disease Control and Prevention (US CDC) website list of flu symptoms include: fever, cough, sore throat, runny or stuffy nose, muscle or body aches, headaches, fatigue (tiredness), vomiting, and diarrhea [25]. In this study, we will include fever, sore throat, cough, headache, vomiting, running nose, and stomach upset, as the clinical symptoms for influenza in schoolchildren.

## Methods

### Data

The study period is the 2005–2006 winter flu season from epidemiological-week (e-week) 50, 2005 to e-week 16, 2006. 1711 study participants were recruited with parental consent from primary schools in four different geographical areas in Taiwan, namely, Taipei City, Changhua, Ilan, and Kinmen Island. One school was chosen from each area, with the exception of Kinmen which had two schools chosen. Most participants were grade 3–5 primary school students, except in one school in Kinmen where students from grade 1–6 were recruited, due to its small sample size. The four areas were chosen owing to their different risk levels for influenza. Taipei City is a metropolis of high-density population with many foreign immigrants and visitors, and hence influenza viruses could be transmitted easily; Kinmen is an off-shore island with frequent traffic to and from the nearby Chinese mainland; Ilan is a suburban county; and Changhua County is a rural area.

Paired serum samples were collected from the students twice, before and after the 2005–2006 influenza season during November 2005 and April 2006, respectively, with the signed informed consent from the student’s parents or guardians. Factors relating to risk or protection factors and demographic information were obtained through questionnaire filled out by student’s parents or guardians, which was collected along with the signed informed consent for the after-season sampling. In addition, questionnaires on influenza-like clinical symptoms (i.e., fever, sore throat, cough, headache, vomiting, running nose, and stomach upset) and whether the children had received influenza vaccination during the study period were collected at the second after-season sampling time. 1062 children remained for the complete study period. The study protocol was approved by the Ethics Committee of the Taipei Hospital. For details of the sampling study and of the study participants, see [26,27].

Each serum specimen contained 3-5 ml of whole blood collected in serum tube and centrifuged at 1,200 rpm/ 10 mins, 4°C within 24 hours to separate RBC and serum. The serum samples were stored at −20°C. Serum samples were treated by RDE (Cambrex) to remove non-specific inhibitors in serum before Haemagglutination-Inhibition (HI) test. Seroconversion is defined as ≧4-fold rise in hemagglutination inhibition antibody (HI Ab) serotiter [28]. Seroprotection is defined as the HI titer ≧40. We compute the geometric mean titer (GMT) of a group of subjects when data analysis is needed to compare the antibody levels between different groups, and a HI titer of less than 10 is assigned a value of 5 for the computation of GMT. The virus strains selected in this study were three human influenza virus vaccine-like strains recommend by the World Health Organization (WHO) in 2005; namely, A/New Caledonia/20/99 (H1N1), A/California/7/2004 (H3N2) and B/ShangHI/361/2002. All vaccine strains were derived from the Taiwan Center for Disease Control and Prevention (TCDC) and grew in Madin-Darby Canine Kidney (MDCK) cells for two passages.

A total of 586 children who had not been vaccinated for influenza in the last 12 months prior to the start of the study are included for this current study. To avoid the confounding effects of existing pre-immunity on seroconversion of the schoolchildren, we only include those children with pre-season HI titer < 40, which totals 255. Demographic characteristics and GMTs of these 255 children are given in Table 1. The seroconversion rate of influenza infection (≧4-fold rise in HI titer) among these 255 schoolchildren for the 3 above-mentioned vaccine strains is given in Table 2.

**Table 1:** Demographic characteristics and geometric mean serotiters

**Characteristics**		**KM**	**CH**	**TP**	**IL**	**Total**
		**n (%)**	**n (%)**	**n (%)**	**n (%)**	**n (%)**
Cohort size		71 (27.84)	60 (23.53)	43 (16.86)	81 (31.76)	255
Gender	Male	31 (43.66)	36 (60.00)	16 (37.21)	41 (50.62)	124 (48.63)
	Female	38 (53.52)	24 (40.00)	22 (51.16)	40 (49.38)	124 (48.63)
	Missing	2 (2.82)	0 (0.00)	5 (11.63)	0 (0.00)	7 (2.75)
Grade	1-3	23 (32.39)	29 (48.33)	19 (44.19)	30 (37.04)	101 (39.61)
	4-6	48 (67.61)	31 (51.67)	24 (55.81)	50 (61.73)	153 (60.00)
	Missing	0 (0.00)	0 (0.00)	0 (0.00)	1 (1.23)	1 (0.39)
GMT (pre-season)		16.94	13.82	13.58	11.57	13.78
GMT (post-season)		41.59	19.32	21.76	25.41	26.67

255 unvaccinated schoolchildren from Taipei City (TP), Changhua (CH), Ilan (IL), and Kinmen (KM) participated in the study.

**Table 2:** Summary table for pathogen-specific seroconversion rates (≧4-fold rise in HI serotiter) of the 255 schoolchildren

**Vaccine strain**	**Seroconversion number (%)**
A/New Caledonia/20/99 (H1N1)	80 (31.37)
A/California/7/2004 (H3N2)	31 (12.17)
B/Shanghai/361/2002 (B)	4 (1.57)

Moreover, it has been reported that, for 2005–2006, of the three vaccine strains only A/New Caledonia/20/99 (H1N1) vaccine strain is found to be antigenically related to the circulating strain in Taiwan [29]. Since the serotesting in this study were carried out with the vaccine strain only, we will focus our study on the seroprotection and seroconversion of A/New Caledonia/20/99 (H1N1) of the 255 unvaccinated children with pre-season HI titer < 40 for H1N1.

### Statistical method

First we utilize the logistic regression model to distinguish the most important symptoms of influenza infection by fitting a logistic regression model to the binary influenza infection outcome in the sample, using binary indicators of the influenza-like symptoms as predictors. Univariate analysis by Fisher exact test and stepwise logistic regression were used to identify the symptoms that influenced the infection during the flu season. The Receiver Operating Characteristic (ROC) curve is used to examine the performance of logistic regression model. We start with a comprehensive model that includes most conceivable and testable factors of an influenza infection. We then exclude covariates with p-values exceeding 0.5. Those covariates with high p-values indicate that they probably contribute more noise than predictive information to the model. Lastly, we implemented the stepwise method for selecting the best possible submodel. Relevant statistical details are given in the Appendix.

The second part of our analysis involves estimating the asymptomatic ratio based on our earlier findings of the most predictive clinical symptoms for influenza infection. We then compute the asymptomatic infection ratios and 95% confidence intervals under these sets of symptoms and investigate the asymptomatic infection ratios with stratified data.

## Results

### Symptoms of influenza infection

A total of 124 of the 1062 children who completed the study reported to have had some symptoms between two samplings. Moreover, 80 children were determined to have seroconverted for A/New Caledonia/20/99 (H1N1), for whom the outcome of univariate analysis of all binary influenza-like symptoms is shown in Table 3, indicating four factors (fever, sore throat, headache, and vomiting) have statistically significant effects on the influenza infection.

**Table 3:** Univariate analysis of influenza-like symptoms for H1N1 (n = 255)

**Symptom**	**H1N1 Seroconversion n = 80 (%)**	**Tested Negative for H1N1 n = 159 (%)**	**P-value**
Fever	53 (66.3%)	36 (22.6%)	<.0001*
Sore Throat	37 (56.9%)	48 (36.9%)	.0094*
Cough	59 (83.1%)	104 (72.7%)	.1246
Headache	31 (50.8%)	26 (21.3%)	<.0001*
Vomiting	17 (32.1%)	11 (9.9%)	.0007*
Running Nose	55 (82.1%)	113 (80.7%)	.8520
Stomach upset	10 (20.0%)	11 (9.8%)	.0824
Demographic variables			
Gender			.1657
Male	34 (43.0%)	82 (53.6%)	
Female	45 (57.0%)	71 (46.4%)	
Grade			.0172
Low (1–3)	39 (48.8%)	52 (32.7%)	
High(4–6)	41 (51.3%)	107 (67.3%)	

*denotes statistically significant at 5% level.

Stepwise multivariate logistic regression analysis is then applied to developing the prediction model. An analysis of multivariate logistic regression, with odds ratio (OR) and 95% confidence interval (CI), is shown in Tables 4 and 5. Clinical symptoms that are significant predictive indicators for influenza infection (Table 4a) include the fever (OR =5.20, 95% CI: 2.30-12.20) and headache (OR = 4.38, 95% CI: 1.36-15.18). Predictive symptoms for influenza infection, with gender and grade added (Table 5b), include the fever (OR = 3.92, 95% CI: 1.67-9.40), headache (OR = 4.90, 95% CI: 1.44-18.05), and vomiting (OR = 5.77, 95% CI: 1.07-36.67).

**Table 4:** Multivariate logistic regression analysis for H1N1

**Symptom**	**OR (95% CI)**	**P-value**
Fever	5.20 (2.30-12.20)	<0.0001*
Sore throat	1.58 (0.59-4.14)	0.353
Cough	2.33 (0.87-6.96)	0.107
Headache	4.38 (1.36-15.18)	0.015*
Vomiting	5.59 (0.98-37.88)	0.060
Running nose	1.09 (0.41-3.06)	0.862
Stomach upset	1.20 (0.09-11.73)	0.881

*denotes statistically significant at 5% level.

**Table 5:** Multivariate logistic regression analysis for H1N1 with gender and grade

**Variables**	**OR (95% CI)**	**P-value**
Fever	3.92 (1.67-9.40)	0.0018*
Sore throat	1.86 (0.68-5.00)	0.2167
Cough	2.25 (0.82-6.86)	0.1309
Headache	4.90 (1.44-18.05)	0.0128*
Vomiting	5.77 (1.07-36.67)	0.0467*
Running nose	0.93 (0.34-2.66)	0.8891
Stomach upset	0.84 (0.07-8.15)	0.8852
Gender (M)	0.66 (0.28-1.55)	0.3420
Grade (H)	0.46 (0.18-1.10)	0.0841

*denotes statistically significant at 5% level.

The most useful prognostic variables for the logistic regression model with a threshold probability of 0.5 are used to predict the patients who are likely to have been infected. Table 6 shows the sensitivity and specificity analyses to assess the prediction power of logistic regression models. In addition, there are other commonly used measures of the performance measures of a prediction model, namely, positive predictive value (PPV), defined as the proportion of patients with predicted infection who are correctly predicted, and negative predictive value (NPV), defined as the proportion of patients with predicted non-infection who are correctly predicted. The last two models, denoted by models (1) and (2) in Table 6, appear to be the best models of symptom predictors for influenza infection.

**Table 6:** Multivariate predictors of influenza infection with PPV, NPV, sensitivity, and specificity analyses with 95% confidence intervals (in parenthesis)

**Symptom**	**PPV**	**NPV**	**Sensitivity**	**Specificity**
Fever	59.6 (51.5-67.1)	82.0 (76.8-86.2)	66.3 (54.8-76.5)	77.4 (70.1-83.6)
Fever + headache	77.4 (61.0-88.3)	75.7 (71.7-79.3)	39.3 (27.1-52.7)	94.3 (88.5-97.7)
Fever + headache + cough	77.8 (59.9-89.1)	75.7 (71.8-79.2)	36.8 (24.4-50.7)	94.9 (89.3-98.1)
Fever + headache + throat	75.0 (60.2-85.6)	79.0 (74.4-82.9)	48.2 (34.7-62.0)	92.4 (86.0-96.5)
Fever + headache + vomiting (model 1)	90.5 (69.7-97.5)	77.9 (73.6-81.6)	39.6 (25.8-54.7)	98.1 (93.2-99.8)
Fever + headache + vomiting + grade (model 2)	67.4 (55.3-77.5)	84.0 (78.0-88.5)	64.6 (49.5-77.8)	85.6 (77.3-91.7)

**Figure 1:**
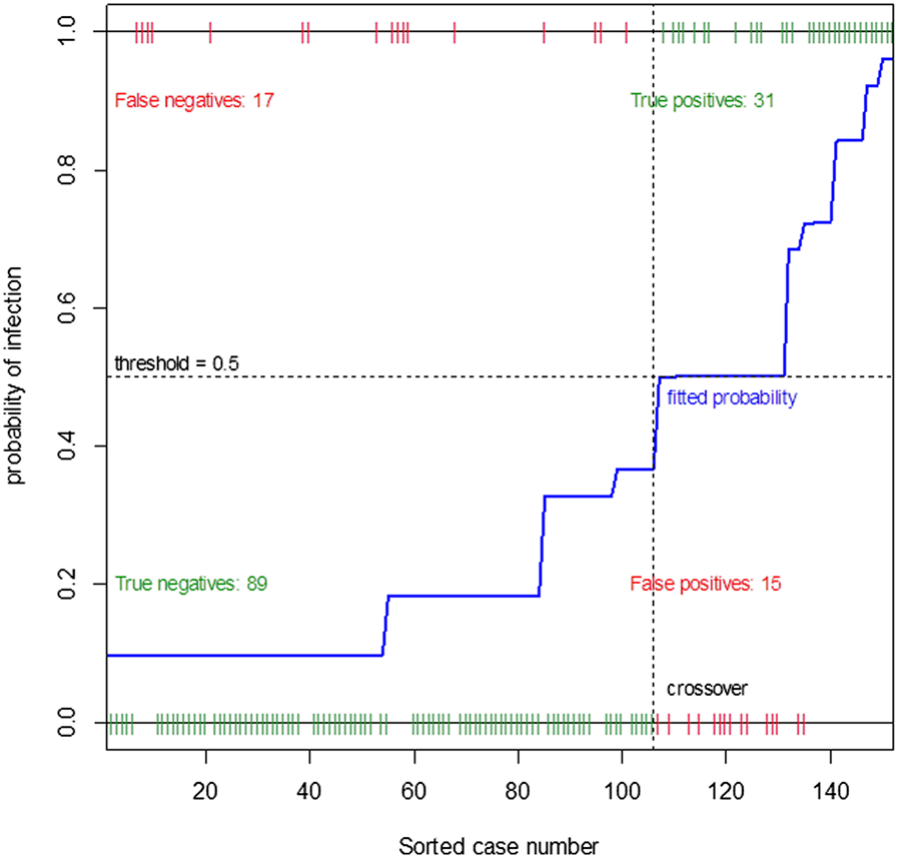
Logistic model of H1N1 influenza infection predicted by fever, headache, vomiting, and grade.

Figure 1 presents a plot of the logistic model of influenza infection as predicted by the four statistically significant symptoms. The fitted probabilities of infection are sorted by probability so that the less probable infections are located to the left and the most probable infections are to the right. The patients were observed either as infection (coding as 1) at the top or no infection (coding as 0) at the bottom. The red ticks represent errors; either false positives or false negatives. Clearly, more false negatives lead to lower sensitivity, whereas less false positives lead to higher specificity. The line of fitted probability is away from the threshold of 0.5, shown as a horizontal dash line. In particular, some patients clustered to the right and to the left are predicted very well. There is a clear difference between symptoms of those with infection and those without.

**Figure 2:**
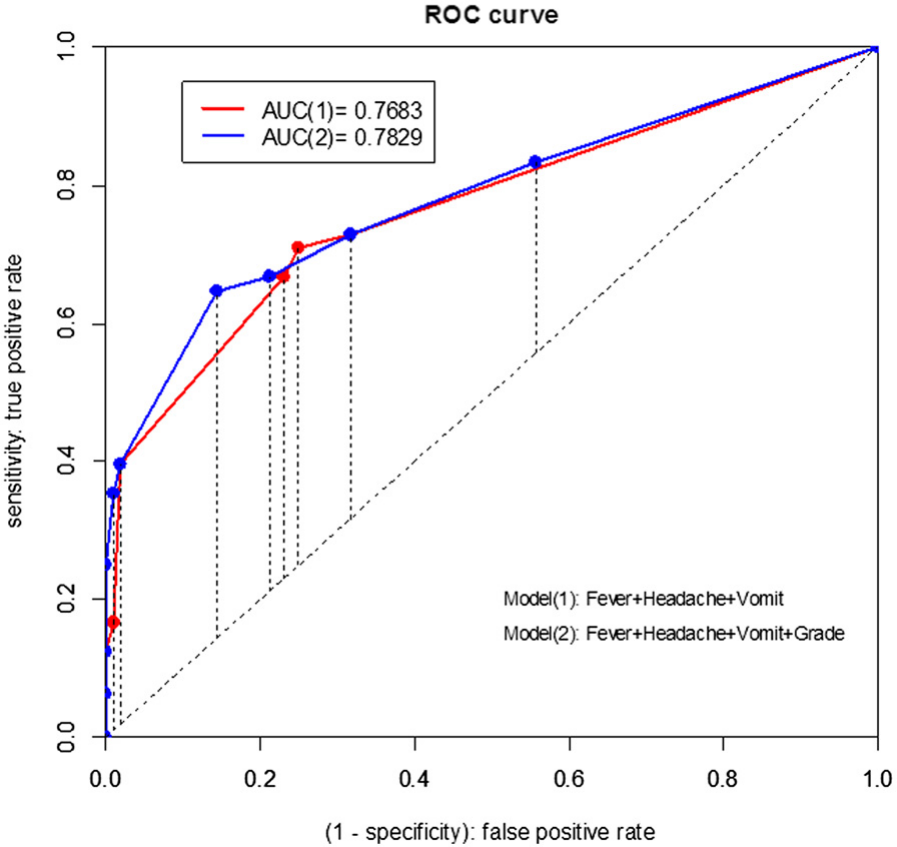
ROC curves of two logistic models (1) and (2) for influenza infection prediction, where the model (1) is a three-variable model based on predictors (fever, headache, and vomiting) and the model (2) is a four-variable model with the grade added to the model (1).

The ROC curve shown in Figure 2 is a plot of the sensitivity of the model prediction against the complement of its specificity at a series of thresholds for a positive outcome to help visualize prediction performance. The further apart is the curve from the diagonal, the more accurate the model is. The area under the ROC curve (AUC) provides an overall measure of classification accuracy of the model, with the value of one representing perfect accuracy. The ROC curve shows a moderate ability to discriminate influenza infection with AUC = 0.75.

### Stratification analysis of influenza infection by age

The total children are stratified by the grades for the logistic regression analysis. The children students are divided into younger children with grade 1–3 and older children with grade 4–6. Table 7 shows the outcomes of a univariate analysis of factors associated with influenza infection according to the stratification criterion. Fever is the most significant risk factor in both groups. Fever (p < 0.0001), cough (p = 0.261), headache (p = 0.018), and vomiting (p = 0.0341) are significant risk factors for the younger schoolchildren; whereas in the older group, fever (p < 0.0001), sore throat (p-value = 0.0125), headache (p-value = 0.010), and vomiting (p = 0.0191) are significant.

**Table 7:** Univariate analysis of influenza-like symptoms for grade stratification

**Symptom**	**Seroconversion for Influenza**	**Tested Negative for Influenza**	**P-value**
Grades 1-3	n = 39	n = 52	
Fever	29 (74.4%)	17 (32.7%)	<.0001
Sore throat	16 (48.5%)	14 (33.3%)	.2370
Cough	31 (86.1%)	30 (63.8%)	.0261*
Headache	16 (48.5%)	6 (13.6%)	.0018*
Vomiting	9 (31.0%)	4 (10.0%)	.0341*
Running nose	23 (76.7%)	41 (87.2%)	.3497
Stomach upset	4 (15.4%)	3 (7.7%)	.4240
Grades 4-6	n = 41	n = 107	
Fever	24 (58.5%)	19 (17.8%)	<.0001*
Sore throat	21 (65.6%)	34 (38.6%)	.0125*
Cough	28 (80.0%)	74 (77.1%)	.8152
Headache	15 (53.6%)	20 (25.6%)	.0100*
Vomiting	8 (33.3%)	7 (9.9%)	.0191*
Running nose	32 (86.5%)	72 (77.4%)	.3327
Stomach upset	6 (25.0%)	8 (11.0%)	.1033

*denotes statistically significant at 5% level.

In the stepwise logistic regression model, the only risk factor that are significantly associated with influenza infection is cough (OR = 4.62, 95% CI: 1.05-65.83) in the grade 1–3 group while in the grade 4–6 group only fever is significant (OR = 4.05, 95% CI: 1.20-13.94), as shown in Table 8.

**Table 8:** Multivariate logistic regression analysis for schoolchildren stratified age/grade

**Age/Grade**	**Symptom**	**OR (95% CI)**	**P-value**
1-3	Fever	2.61 (0.68-10.05)	.1628
	Sore throat	0.90 (0.15-4.47)	.8945
	Cough	4.62 (1.05-65.83)	.0464*
	Headache	19.78 (0.77-509.94)	.0718
	Vomiting	8.12 (0.53-589.51)	.1615
	Running nose	0.45 (0.06-2.04)	.3157
	Stomach upset	0.45 (0.004-7.74)	.6383
4-6	Fever	4.05 (1.20-13.94)	.0237*
	Sore throat	2.67 (0.74-9.55)	.1280
	Cough	1.37 (0.35-6.00)	.6592
	Headache	2.88 (0.66-12.83)	.1551
	Vomiting	4.30 (0.44-52.00)	.2152
	Running nose	1.85 (0.48-8.36)	.3894
	Stomach upset	1.64 (0.04-85.14)	.7934

*denotes statistically significant at 5% level.

**Figure 3:**
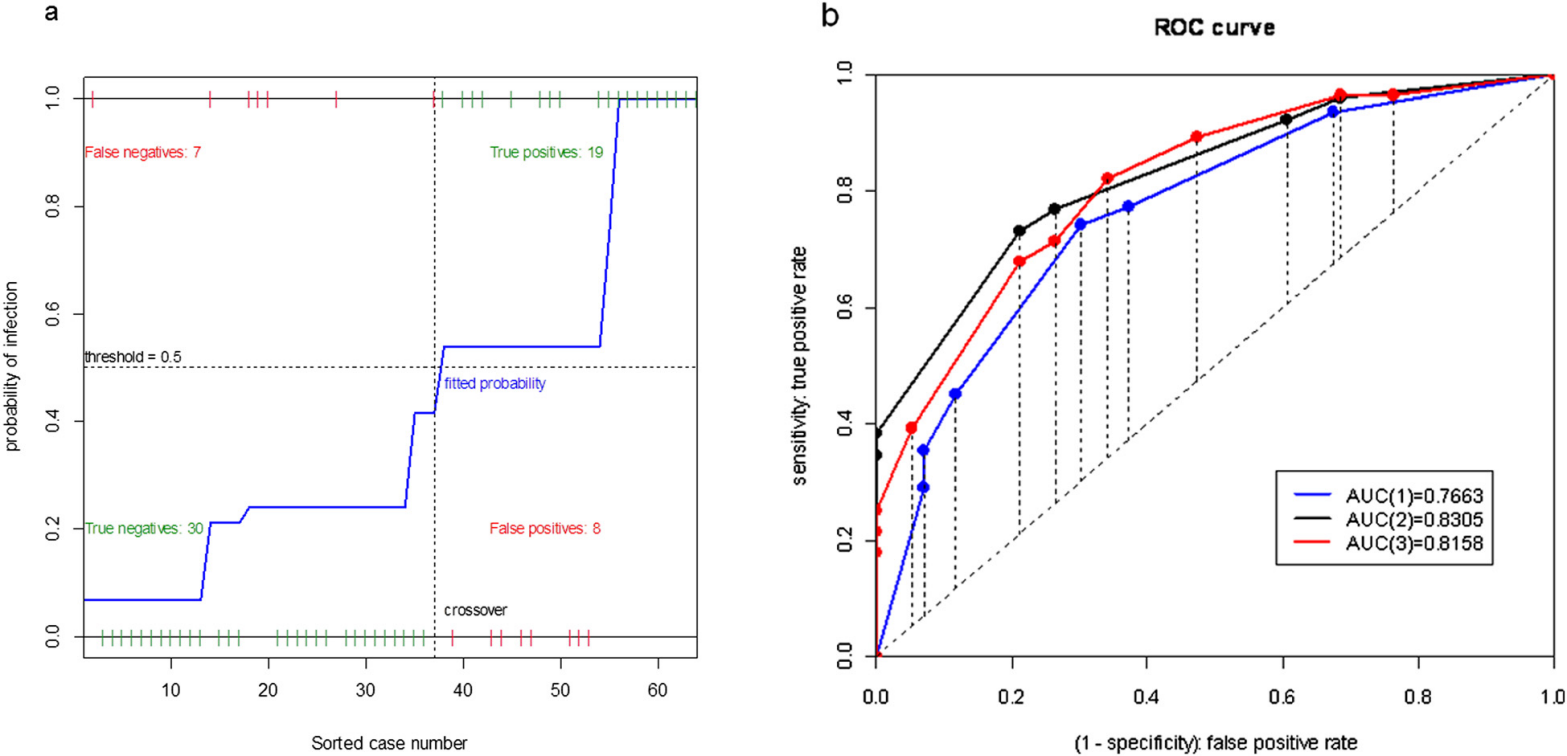
**The plots of the logistic model and the ROC curves for Tables**8**,**9**for Younger Schoolchildren of grades 1–3. (a)** Logistic model of H1N1 influenza infection predicted by fever, headache, vomiting, and cough. **(b)** ROC curves of combination of symptoms for influenza infection prediction in last 3 rows of younger schoolchildren of grades 1–3 in Table 9, denoted respectively by (1), (2), and (3).

**Figure 4:**
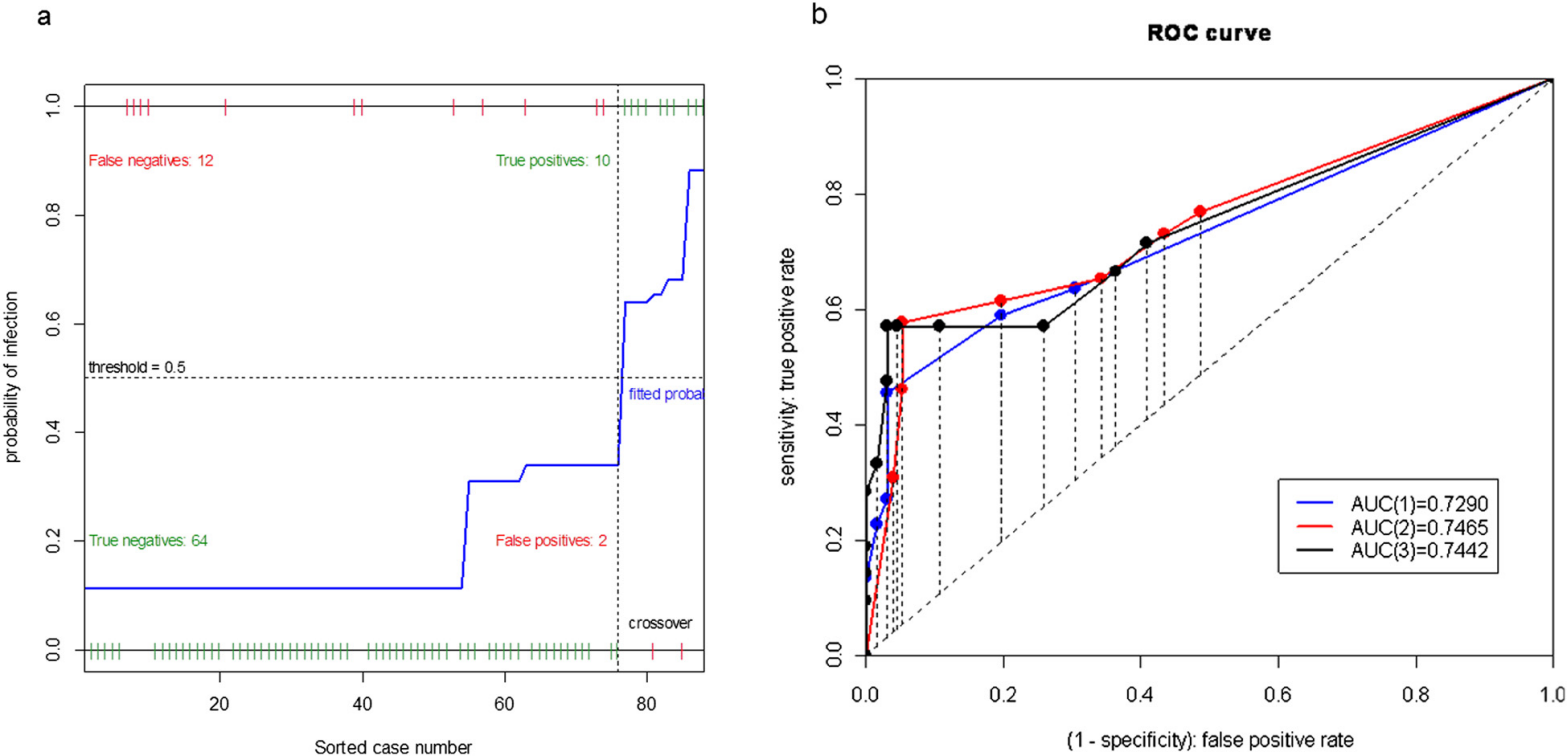
**The plots of the logistic model and the ROC curves for Tables**8**,**9**for Older Schoolchildren of grades 4–6. (a)** Logistic model of H1N1 influenza infection predicted by fever, headache, vomiting, and sore-throat. **(b)** ROC curves of combination of symptoms for influenza infection prediction in last 3 rows of Table 9, denoted respectively by (1), (2), and (3).

In Table 9, we compare the results of applying different combinations of risk factors to the younger (grades 1–3) and elder (grades 4–6) groups. That is, we compare PPV, NPV, Sensitivity, and Specificity using two significant risk factors in younger group with using one significant risk factor in elder group. The latter outperforms the former in terms of sensitivity and specificity. Overall, both models reveal high specificity and low sensitivity. However, this difference is possibly due to a proportion of asymptomatic infection. The plots of the logistic model and the ROC curves for Tables 8, 9 are given in Figures 3, 4. Note that in Figure 3(b), we provide the ROC curves for combination of symptoms in last 3 rows of influenza infection prediction for younger schoolchildren of grades 1–3 in Table 9, denoted respectively by (1), (2), and (3). In Figure 4(b), ROC curves are given for combination of symptoms in last 3 rows of Table 9, again denoted respectively by (1), (2), and (3), for influenza infection prediction for older schoolchildren of grades 4–6.

**Table 9:** Multivariate predictors of influenza infection

**Grade**	**Symptom**	**PPV**	**NPV**	**Sensitivity**	**Specificity**
1-3	Fever	63.0 (52.6-72.4)	77.8 (66.5-86.1)	74.4 (57.9-87.0)	67.3 (52.9-79.7)
	Fever + cough	67.7 (54.1-78.8)	73.5 (63.6-81.5)	63.9 (46.2-79.2)	76.6 (62.0-87.7)
	Fever + headache	72.7 (53.9-85.9)	69.1 (61.1-76.1)	48.5 (30.8-66.5)	86.4 (72.6-94.8)
	Fever + vomiting	63.6 (50.9-74.7)	77.8 (65.2-86.7)	72.4 (52.8-87.3)	70.0 (53.5-83.4)
	Fever + cough + vomiting	69.0 (54.4-80.6)	76.9 (65.4-85.5)	69.0 (49.2-84.7)	76.9 (60.7-88.9)
	Fever + cough + headache	63.9 (51.8-74.5)	79.0 (66.7-87.5)	74.2 (55.4-88.1)	69.8 (53.9-82.8)
	Fever + cough + vomiting + headache	70.4 (55.1-82.1)	81.1 (69.0-89.2)	73.1 (52.2-88.4)	79.0 (62.7-90.4)
	Fever + cough + vomiting + sore throat	66.7 (52.8-78.2)	77.8 (65.4-86.6)	71.4 (51.3-86.8)	73.7 (56.9-86.6)
4-6	Fever	55.8 (43.8-67.2)	83.8 (78.1-88.3)	58.5 (42.1-73.7)	82.3 (73.7-89.0)
	Fever + cough	53.6 (38.0-68.5)	80.6 (75.5-84.8)	42.9 (76.3-60.6)	86.5 (78.0-92.6)
	Fever + headache	73.3 (48.8-88.8)	81.3 (76.3-85.5)	39.3 (21.5-59.4)	94.9 (87.4-98.6)
	Fever + vomiting	85.7 (43.2-97.9)	79.6 (75.5-83.1)	25.0 (9.8-46.7)	98.6 (92.4-100)
	Fever + headache + vomiting	83.3 (54.2-95.5)	84.2 (78.4-88.7)	45.5 (24.4-67.8)	97.0 (89.5-99.6)
	Fever + headache + sore throat	79.0 (57.8-91.1)	86.8 (80.6-91.1)	57.7 (36.9-76.6)	94.7 (87.1-98.5)

PPV, NPV, Sensitivity, and Specificity Analyses for Schoolchildren are stratified by grade with 95% confidence intervals (in parenthesis).

### Asymptomatic ratio

We estimate the asymptomatic ratios (Table 10) based on the symptoms (or combination of symptoms) with the highest correlation from earlier analysis. We also obtain the asymptomatic ratio based on no symptoms, as this criterion is often used in literature [6]. Moreover, asymptomatic ratios based on the criteria for influenza-like-illness (ILI) used by US-CDC (i.e., fever + (cough or sore throat)), and TCDC (i.e., fever + (cough or sore throat or running nose) + ( headache or pain or fatigue)) are also provided.

**Table 10:** Asymptomatic ratios (in%) with 95% confidence intervals based on combination of symptoms

**Symptoms**	**Asymptomatic ratio (%)**
Fever	65.1 (54.7-75.6)
Fever + cough	70.5 (60.6-80.5)
Fever + (cough or vomiting)	67.2 (56.9-77.5)
Fever + (cough or headache)	63.8 (53.2-74.3)
Fever + (cough or nose)	63.9 (53.3-74.4)
Fever + (cough or vomiting or headache)	61.2 (50.5-71.9)
Fever + sore throat	77.9 (68.8-87.0)
Fever + ( sore throat or vomiting)	73.7 (64.1-83.4)
Fever + ( sore throat or headache)	71.1 (61.1-81.0)
Fever + ( sore throat or nose)	66.7 (56.3-77.0)
Fever + ( sore throat or vomiting or headache)	68.9 (58.8-79.1)
Any symptom	55.6 (44.7-66.4)
Fever + (cough or sore throat)*	66.9 (56.6-77.2)
Fever + (cough or sore throat or nose) + (headache or pain or fatigue)^#^	73.0 (63.3-82.8)

*denotes US-CDC criteria for ILI; ^#^denotes TCDC criteria for ILI.

We also consider asymptomatic ratios stratified by location, gender, and age/grade. Asymptomatic ratios stratified by the 4 areas are given in Table 11. For all cases, stratified asymptomatic ratios in Taipei City are always significantly smaller than that of the other locations. The corresponding estimates for the asymptomatic ratios stratified by gender and age are not statistically significantly different, and hence the details are omitted here.

**Table 11:** Asymptomatic ratios with 95% confidence interval based on combination of symptoms stratified by location: Kinmen (KM), Changhua (CH), Ilan (IL), and Taipei City (TP)

**Symptoms**	**Asymptomatic ratio stratified by area (%)**				**P-value**^**1**^
	**KM**	**CH**	**IL**	**TP**	
Fever	64.8 (48.3-81.4)	73.9 (43.5-100)	84.9 (72.2-97.5)	45.2 (12.7-77.8)	0.0033
Fever + cough	76.5 (61.8-91.2)	76.2 (46.7-100)	87.9 (76.4-99.4)	29.2 (0.0-58.9)	<.0001
Fever + (cough or vomiting)	71.4 (55.8-87.1)	76.2 (46.7-100)	87.9 (76.4-99.4)	10.5 (0.0-30.6)	<.0001
Fever + (cough or headache)	64.6 (48.0-81.2)	76.2 (46.7-100)	87.9 (76.4-99.4)	20.0 (0.0-46.1)	<.0001
Fever + (cough or nose)	66.7 (50.3-83.0)	76.2 (46.7-100)	84.9 (72.2-97.5)	20.0 (0.0-46.1)	<.0001
Fever + (cough or vomiting or headache)	62.5 (45.7-79.3)	76.2 (46.7-100)	87.9 (76.4-99.4)	9.1 (0.0-27.9)	<.0001
Fever + sore throat	82.0 (68.7-95.3)	100.0 (−)	87.9 (76.4-99.4)	22.2 (0.0-49.4)	<.0001
Fever + ( sore throat or vomiting)	75.5 (60.6-90.4)	100.0 (−)	87.9 (76.4-99.4)	0.0 (−)	<.0001
Fever + ( sore throat or headache)	68.8 (52.7-84.8)	100.0 (−)	87.9 (76.4-99.4)	15.8 (0.0-39.6)	<.0001
Fever + ( sore throat or nose)	72.0 (56.4-87.6)	81.0 (53.7-100)	84.9 (72.2-97.5)	13.6 (0.0-36.1)	<.0001
Fever + ( sore throat or vomiting or headache)	66.7 (50.3-83.0)	100.0 (−)	87.9 (76.4-99.4)	0.0 (−)	<.0001
Any symptom	39.1 (22.2-56.0)	65.0 (31.9-98.1)	72.7 (57.1-88.4)	0.0 (−)	<.0001
Fever + (cough or sore throat)*	74.0 (58.8-89.2)	76.2 (46.7-100)	84.9 (72.2-97.5)	17.4 (0.0-42.2)	<.0001
Fever + (cough or sore throat or nose) + (headache or pain or tired)^#^	72.9 (57.5-88.3)	90.5 (70.1-100)	90.9 (80.8-100)	0.0 (−)	<.0001

*denotes US-CDC criteria for ILI; ^#^denotes TCDC criteria for ILI.

^1^P-value from chi-square test.

## Conclusions and discussions

The asymptomatic influenza ratio for schoolchildren estimated in this study is found to be considerably higher than that of all age groups in previous studies [6]. There are several possible explanations. First, there are very few studies in the past that focused on children alone and our study indicates that age-specific difference in asymptomatic ratio can be significant. Moreover, our community-based study includes children recruited from the community where pre-immunity exists, perhaps at a substantial level, which might also lead to milder symptoms. However, in this study we have excluded all children with prior seroprotection of HI titer greater than or equal 40 to avoid this confounding factor. Finally, our study is confined to that of seasonal H1N1 infection. It has been shown in a comparative study [26] of pathogen-specific asymptomatic ratio for influenza based on this same data set but using having fever or body aches + headache as the criteria for symptoms, that the asymptomatic ratio for seasonal H1N1 (75%) is higher than that of seasonal H3N2 (65%), perhaps reflecting more frequent infection of H1N1 during past influenza seasons on the population-level. Note that in [27], children with high pre-season HI titers were not excluded from their analysis which contributes to a higher asymptomatic ratio.

We note that there has been a significant body of literature on the sensitivity and specificity of selected ILI symptoms to actual influenza infection (e.g., [22,24]). In this study, we have focused primarily on the logistic regression model, commonly used to analyze medical prognostication model. This model has the advantage of easy explanations for the model parameters in practice. Although we use a stepwise logistic regression to assist us in developing the prediction model in this study, the numerous disadvantages of stepwise selection are well known and discussed within statistical literatures. The principal drawbacks of stepwise selection include biases in parameter estimation and reported p-value, inconsistencies among model selection algorithms, an issue of multiple hypotheses testing, and the possibility of missing the optimal model. To overcome these obstacles, we implement the stepwise regression in conjunction with considering all possible subsets of the same number of factors as in the stepwise solution to examine whether some other subsets of factors might be better. In addition, the AIC criterion, the ROC, and clinical knowledge are utilized to determine the best possible submodel. We note that some more recently proposed regularized regression technique, such as the least-angle regression (LARS-Lasso) algorithm [30], might also be useful to identify the set of symptoms most predictive of an influenza infection.

The asymptomatic ratio among schoolchildren in Taipei City, the only urban city in our study with markedly higher level of education among parents compared to other rural areas, is significantly smaller than that of the other rural locations. No other significant regional difference in the respective asymptomatic ratios is observed, perhaps partly due to stratification resulting in smaller cohort sizes. The asymptomatic ratios of younger schoolchildren of grades 1–3 are slightly higher than those of older children of grades 4–6, indicating the various factors such as community setting (urban vs. rural) and age which might affect the asymptomatic ratio.

Children are known to have higher infection rates than adults and high viral transmission with clustering cases with higher influenza virus isolation rate often found in children when compared with adults. Therefore, schoolchildren are vectors in influenza epidemics. One US study indicates that ILI cases increase economic burden among households with school-aged children and lead to more school and workdays lost [31]. When vaccinating those at greatest risk of mortality becomes impractical (if, e.g., medical care were relatively inaccessible) or inefficient (if, e.g., immune responses were deficient), targeting those most likely to expose them might be preferable [32]. One study comparing influenza mortality among elderly Japanese when children were and were not vaccinated suggests infected children pose a risk to others [33], including elderly people, who however also may be infected by intermediates. Numerous US experiences (as summarized in [34]) are also consistent with this conclusion, although the issue has become somewhat controversial following the recent publication of age-specific timing of lab confirmed influenza infections indicating slight age-specific differences in the timing of infection [15].

The asymptomatic ratio relates to the likely success of public health interventions such as the ‘stay home if you’re sick’ message. Furthermore, it is important from the perspective of public health interventions to clarify the symptoms. For example, the PPV and specificity of ‘runny nose’ or ‘cough’ in the absence of any other symptoms would be helpful in defining an appropriate list of symptoms. While predicting the ultimate success of the intervention is a modeling issue, providing an evidence basis and discussion of symptoms as predictors of influenza links directly to clarification of the public health message.

Estimate of the symptomatic ratio of infected children for seasonal H1N1 is important for modeling studies aimed to provide reasonable quantification of the impact of asymptomatic infective children who may be capable of spreading pathogens to others in a seasonal or pandemic epidemic. Ascertainment of the role of children in spread of influenza, including asymptomatic infections, and its interventions is of public health importance in post-pandemic influenza seasons.

## Appendix

Statistical details

Univariate analysis by Fisher exact test and stepwise logistic regression were used to identify the symptoms that influenced the influenza infection during the flu season. Logistic regression models provide odds-ratio estimations and predicted risk of the infection given a set of risk factors, as well as allow adjustment for confounders. (Given a set of risk factors, logistic regression analyses, after adjustment for confounders, provide odds ratio estimates and predicted risk of infection.) Stepwise multivariate logistic regression analysis was performed to identify significant prognostic factors associated with the influenza infection. The logistic regression model provides the estimated probability of infection for a particular patient with symptom variables {X_1_, *X*_2_,…, X_k_}. This probability is equal to y = 1/(1 + e^-z^), where z = β_0_ + β_1_ X_1_ + β_2_*X*_2_ + … + β_k_ X_k_, and e is the base value of natural logarithm. The resulting logistic regression model was made based on the forward stepwise model selection procedure and after further investigating the two-way interactions between the predictors. Receiver operating characteristic (ROC) curves were constructed and the area under an ROC curve (AUC, also known as the c-statistic) provides an overall assessment of prediction performance. All tests were two-sided, and a p-value of <0.05 are considered to be statistically significant.

## Competing interests

The authors declare that they have no competing interests.

## Authors’ contributions

YHH conceived and coordinated the study, and wrote the first draft. CYL and CCK collected the data. CAT and JWC carried out the analysis. YHH, CAT, JWC, CCK, DYC, and KFC participated in the study and the interpretation of study findings. CCK and DYC participated in the writing of the manuscript. All authors have read and approved the final manuscript.

## Pre-publication history

The pre-publication history for this paper can be accessed here:

http://www.biomedcentral.com/1471-2334/14/80/prepub
